# Association of Particulate Matter from Cooking Oil Fumes with Heart Rate Variability and Oxidative Stress

**DOI:** 10.3390/antiox10081323

**Published:** 2021-08-23

**Authors:** Chang-Chuan Chan, Lian-Yu Lin, Ching-Huang Lai, Kai-Jen Chuang, Ming-Tsang Wu, Chih-Hong Pan

**Affiliations:** 1 Institute of Environmental and Occupational Health Sciences, College of Public Health, National Taiwan University, Taipei 10055, Taiwan; ccchan@ntu.edu.tw; 2 Department of Internal Medicine, College of Medicine, National Taiwan University Hospital, Taipei 10050, Taiwan; hspenos@gmail.com; 3 School of Public Health, National Defense Medical Center, Taipei 11490, Taiwan; lgh@ndmctsgh.edu.tw; 4 School of Public Health, College of Public Health and Nutrition, Taipei Medical University, Taipei 11490, Taiwan; kjc@tmu.edu.tw; 5 Department of Public Health, School of Medicine, College of Medicine, Taipei Medical University, Taipei 11490, Taiwan; 6 Department of Public Health, College of Health Sciences, Kaohsiung Medical University, 100 Shih-Chuan 1st Road, Kaohsiung 80787, Taiwan; mingtsangwu@mail.cmu.edu.tw; 7 Research Center for Environmental Medicine, Kaohsiung Medical University, 100 ShihChuan 1st Road, Kaohsiung 87087, Taiwan; 8 Department of Family Medicine, Kaohsiung Medical University Hospital, Kaohsiung Medical University, 100, Tzyou 1st Road, Kaohsiung 80787, Taiwan; 9 Graduate Institute of Clinical Medicine, College of Medicine, Kaohsiung Medical University, Kaohsiung 80787, Taiwan; 10 Institute of Labor, Occupational Safety and Health, Ministry of Labor, New Taipei City 22143, Taiwan

**Keywords:** cooking oil fumes, particulate matter, polycyclic aromatic hydrocarbons, heart rate variability, oxidative stress

## Abstract

Many studies have reported various cardiovascular autonomic responses to ambient particulate matter (PM) pollution, but few have reported such responses to occupational PM exposures. Even fewer have demonstrated a relationship between PM pollution and oxidative stress in humans. This panel study evaluates the association between occupational exposure to PM in cooking oil fumes (COFs), and changes in both heart rate variability (HRV) and oxidative stress responses in 54 male Chinese cooks. Linear mixed-effects regression models were adopted to estimate the strength of the association between PM and HRV. Participants’ pre- and post-workshift urine samples were analyzed for 8-hydroxy-2′-deoxyguanosine (8-OHdG) and malondialdehyde (MDA). Exposure to PM in COFs from 15 min to 2 h were associated with a decrease in HRV and an increase in heart rate among cooks. The urinary 8-OHdG levels of cooks were significantly elevated after workshift exposure to COFs. The levels of PM_2.5_, PM_1.0_, and particulate benzo(a)pyrene in COFs were all positively correlated with cross-workshift urinary 8-OHdG levels. Furthermore, the levels of benzo(a)pyrene in COFs were positively correlated with cross-workshift urinary MDA levels. The effects of COFs on HRV were independent of cross-workshift urinary 8-OHdG levels. Exposure to COFs leads to disturbed autonomic function and an increased risk of oxidative DNA injury among cooks in Chinese restaurants.

## 1. Introduction

Many studies have investigated the association between ambient particulate matter (PM) of various sizes and heart rate variability (HRV) among susceptible populations as well as healthy young people. Most, but not all, have observed a negative association between ambient PM exposure and at least one measure of HRV at various time lags from a few minutes up to 48 h [1,2,3,4,5,6]. One study, however, revealed a positive association between PM and r-MSSD [7]. The HRV reduction was also reported to be associated with PM exposure in the workplace, such as suffered by boilermakers [8] and troopers [9]. In contrast, few studies have demonstrated a relationship between elevated PM levels and increased oxidative stress in humans, partly due to difficulties in measuring and interpreting measures of oxidative stress. One recent study noted that the elevation of oxidative stress marker 8-hydroxy-2′-deoxyguanosine (8-OHdG) level was associated with an increase in nitrate and sulfate levels in urban air pollution [10].

Cooks are occupationally exposed to the hazards of cooking oil fumes (COFs) of which PM and polycyclic aromatic hydrocarbons (PAHs) are important components [11]. Exposure to COFs promotes the induction of lipid peroxidation of lung epithelial cells in vitro [12]. Studies have also demonstrated associations between lipid peroxidation and atherosclerosis [13], aging [14], rheumatoid arthritis [15], diabetes mellitus [16], and cancer [17]. Malondialdehyde (MDA), one of the stable aldehydic products of lipid peroxidation that are present in biological samples such as urine, hair or blood, reflects the global oxidative status of the human body [18,19]. A human observational study has verified that carcinogenic PAHs, such as benzo(a)pyrene (BaP) in COFs, can generate reactive oxygen species (ROS) via cytochrome P-450 A1 during the metabolic process [20]. Urinary 8-OHdG is a biomarker of oxidative stress in DNA and has been adopted to evaluate the extent of repair of ROS-induced DNA damage in both clinical and occupational fields [21,22].

One study reported that cooks had higher oxidative stress levels than housewives and students [23], but no study has ever investigated the effects of COFs on heart rate variability. This panel study of Chinese cooks was designed to explore the effects of PM mass and PAHs in COFs on two markers of oxidative stress, 8-OHdG and MDA. The influence of PM mass in COFs on HRV was considered and whether pre-exposure baseline oxidative stress levels modified the effects of PM exposure on HRV was determined.

## 2. Materials and Methods

### 2.1. Study Subjects 

This panel study continuously and simultaneously monitored changes in PM concentrations and HRV in study subjects during workdays. Biological monitoring was performed to evaluate oxidative stress throughout the workday. The study population comprised 54 male cooks who were monitored while working in 16 Chinese restaurants in Taiwan. A questionnaire was used to collect information on age, height, weight, type of job, health conditions, and life style. Smoking was not allowed in the kitchens and dining areas since all restaurants were designated as non-smoking areas, according to anti-smoking regulations. The Institute Review Board of the Kaohsiung Medical University Hospital approved this study. Informed consent was obtained from all subjects. 

### 2.2. Continuous Holter Monitoring

Continuous ambulatory electrocardiography (ECG) monitoring of each subject was performed using a three-channel digital ambulatory ECG recorder (Aria Pacer model; Del Mar Renolds Medical, Inc., Irvine, CA, USA). ECG monitoring data were analyzed using a Delmar Holter Software, version 2.0 analysis system. The ECG wave complexes (QRS) were automatically classified and manually confirmed to be a normal sinus rhythm, arterial or ventricular premature beats or noise by comparison with the adjacent QRS morphologic features. The normal-to-normal (NN) intervals were determined from the adjacent normal sinus beats. The NN-interval time series was then transferred to a personal computer and post-processed using a program written in MATLAB (version 5.2; MathWorks Inc., Natick, MA, USA). The missing intervals of the raw NN data were linearly interpolated and the data in these intervals were resampled at 4 Hz using the Ron-Berger method. The HRV analysis was performed on 5-min NN intervals. Time-domain calculations of HRV, including SDNN and r-MSDD were made. Furthermore, frequency-domain measurements of HRV, at low frequency (LF) (0.04–0.15 Hz) and high frequency (HF) (0.15–0.40 Hz), were calculated using Welch’s averaged periodogram of NN intervals [24]. To evaluate the effect of PM in COFs and to eliminate any effect of sleep on HRV, Holter monitoring was conducted while the subjects worked between 09:00 and 21:00 to obtain data. 

### 2.3. Particulate Matter

Each study subject underwent continuous, personal PM_10_, PM_2.5_, and PM_1.0_ monitoring using a DUST-check portable dust monitor, Model 1.108 (Grimm Labortechnik Ltd., Ainring, Germany), which measured mass concentration and temperature every minute. The dust monitor was placed near the breathing zone of each subject and was worn throughout the workday. Collocated Rupprecht and Patashnick 1400a tapered element oscillating microbalance (TEOM) samplers (Thermo Electron Corporation, East Greenbush, NY, USA) were used to calibrate the mass concentrations of PM_10_, PM_2.5_, and PM_1.0_ that were measured by the DUST-check monitor in a previous study. Concurrent PM measurements revealed a strong association between the date collected by these two monitors for all three size fractions: PM_10_ (r^2^ = 0.91), PM_2.5_ (r^2^ = 0.90), and PM_1.0_ (r^2^ = 0.80) [25]. 

### 2.4. Particulate PAHs

Particulate PAH samples were collected and HRV and biological monitoring were performed on the same day. Particulate PAHs in the workplace were collected using IOM (Institute of Occupational Medicine, England) samplers with glass fiber filters (diameter: 25 mm, pore size: 0.7 μm) at a flow rate of 2.0 L/min. The samplers were placed near each worker’s breathing zone and worn throughout the workday. The personal particulate PAHs samples were analyzed for five PAHs species—pyrene, benzo(k)fluoranthene (BkF), benzo(a)pyrene (BaP), benzo(ghi)perylene (Bghip), and dibenzo(a,e)pyrene (DBaeP)—using a high-performance liquid chromatography (HPLC) method, which has been described elsewhere [26]. The detection limits were obtained by performing seven repeated analyses of the lowest standards for each PAH species. The coefficient of variation among these repeated analyses was less than 2% for all five PAHs. The detection limits were 0.28 pg of pyrene, 0.72 pg of BkF, 0.28 pg of BaP, 0.63 pg of Bghip, and 0.43 pg of DBaeP.

### 2.5. Urinary 8-OHdG and MDA

Urine samples were collected pre- and post-workshift on Friday morning after the participants had worked for 4 days (Monday through Thursday). Urinary 8-OHdG was analyzed by HPLC/MS/MS, which has been described elsewhere [27]. A detection limit of 5.7 ng/L was obtained from seven repeated analyses of deionized water. The coefficients of variation were less than 5% in inter-day and intra-day tests. 

Urinary MDA concentrations were measured by HPLC, as has been described elsewhere [26]. The within-run and run-to-run precisions of MDA in urine were evaluated. A detection limit of 0.06 μg/L was obtained from seven repeated analyses of deionized water and the coefficient of variation among the repeated analyses was below 10%. 

Each individual’s urinary 8-OHdG and MDA levels was corrected based on the urine creatinine values, which was determined using an automated method based on the Jaffe reaction [28].

### 2.6. Statistical Methods 

Linear mixed effect regression models were used to estimate the effects of PM levels on log10-transformed HR and HRV measurements, by applying general additive procedures. Mixed-effects models have the advantage of allowing the variation of variables that are invariant in fixed-effects models while accounting for differences among individuals using random-effects models. The subjects’ age, years as a cook, BMI, and exposures to cigarette smoke were the time-invariant variables, and PM levels, temperature, HR, and HRV were the time-varying variables. The exposure variables in the models were 15 min to 4 h moving averages of PM. The time courses of the PM exposures were studied only up to 4-h moving averages since the number of available data substantially decreased as the interval of the moving average increased above 5 h. The association between PM and HRV was further evaluated by controlling HR in the model. Modification of the effect of PM on HRV by baseline oxidative stress and smoking status were evaluated by implementing a linear mixed-effects model with and without adjusting the urinary 8-OHdG levels and smoking status, respectively. To test the hypothesis that PM reduces HRV through oxidative stress, the pollution estimates with and without a cross-shift change in urinary 8-OHdG levels, which were the log-transformed differences between pre-work and post-work concentrations, were considered. Both post-workshift and cross-shift changes in oxidative stress levels are treated as outcome variables in our analyses. Paired t-tests were performed to compare pre-workshift and post-workshift levels after log-transformation. Linear mixed-effects models were then adopted to study how particulate PAHs and PM affect cross-shift changes in urinary 8-OHdG and MDA concentrations, after adjustments were made for age, BMI, years as a cook, and cigarette smoking. A level for statistical significance of α = 0.05 was adopted in all tests. All statistical analyses were performed using the S-PLUS 2000 program (MathSoft Inc., Cambridge, MA, USA).

## 3. Results

### 3.1. Personal Characteristics and Environmental Exposures

Table 1 summarizes the personal characteristics and environmental exposures of the study participants. The study population comprised 54 Taiwanese male cooks with a mean age of 33.6 years (SD = 10.5 years), and BMI from 17.6 to 32.9 (mean ± SD, 23.2 ± 3.7); 10 of them were classified as obese subjects (BMI ≥ 27); almost half (48.1%) were smokers. They had spent an average of 13.4 years (SD = 10.6 years) as cooks. The HRV indices were means over all participants during the 12-h monitoring period. The mean heart rate was 91.3 ± 10.2 beats per minute (bpm). 

The 5-min mean PM_10_, PM_2.5_, and PM_1.0_ were 72.8 ± 134.7 μg/m^3^ (range: 1.9–2481.9 μg/m^3^), 49.7 ± 56.2 μg/m^3^ (range: 1.5–1168.3 μg/m^3^), and 37.3 ± 36.2 μg/m^3^ (range: 1.2–532.3 μg/m^3^), respectively. However, PM levels fluctuated widely, as specified by the large standard deviations in the mass concentration statistics and as indicated by large within- and between-subject variations in PM exposure over the study period. The 5-min ambient temperature during the periods of monitoring of the 54 cooks ranged from 16.0 to 36.9 °C. The concentrations of five PAHs were from 1.7 ± 3.0 ng/m^3^ to 11.4 ± 20.1 ng/m^3^. All PM and PAH data were skewed to the right, as their median values were lower than the means.

### 3.2. PM Effects on HR and HRV

Table 2 presents significant effects of PM_10_, PM_2.5_, PM_1.0_ on HR and time-domain HRV based on 15-min to 2-h moving averages. The results of PM exposures averaged over 3-h or 4-h averages are not shown in the table since they exhibited no significant association. The increase in HR and decrease in time-domain HRV were significantly associated with an increase in PM_2.5_ and PM_1.0_ exposures based on 2-h averages, and with an increase in PM_10_ exposures based on 1-h averages. An interquartile range (IQR) increase in PM exposure was associated with a 0.7 to 2.7% increase in HR and a 0.7 to 4.5% decrease in time-domain HRV. BMI was significantly and negatively associated with time domain HRV indices and the number of years as a cook was marginally associated with time-domain HRV indices. 

The effects of PM_2.5_ and PM_1.0_ exposure on the frequency-domain HRV of LF and HF were similar to those revealed by the time-domain HRV indices, as shown in Table 3. The 5-min LF and HF exhibited a statistically significantly negative relationship with PM_2.5_ and PM_1.0_ exposures based on 15-min to 2-h moving averages; statistically insignificant results were observed for PM_10_. The mean decrease in LF and HF for every IQR increase in PM_2.5_ and PM_1.0_, respectively, ranged from 0.71 to 6.86%. Again, BMI was the confounder that was negatively associated with frequency-domain HRV indices.

**Figure 1:**
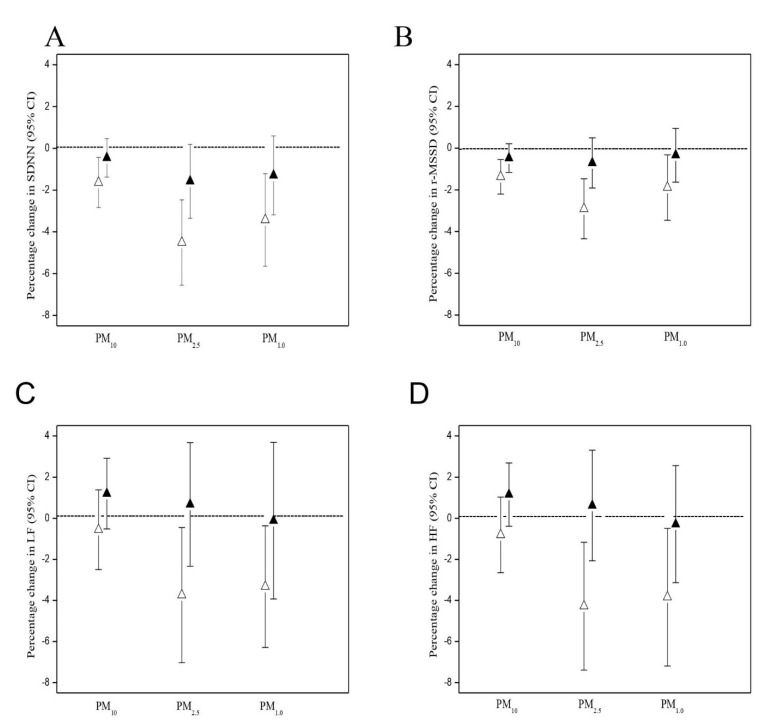
Comparisons of effects of 1-h moving average PM exposure on heart rate variability (HRV) among 54 workers with (solid triangles) and without (open triangles) adjustment of heart rate as estimated using linear mixed effects models. (**A**) Effects of 1-h moving average PM exposure on SDNN, (**B**) Effects of 1-h moving average PM exposure on r-MSDD, (**C**) Effects of 1-h moving average PM exposure on LF, (**D**) Effects of 1-h moving average PM exposure on HF. Models were adjusted for age, BMI, smoking, years as a cook, and ambient temperature.

Figure 1 shows the effects of an interquartile range increase in PM exposures on changes in HRV with the adjustment of HR in the mixed-effect models. The PM of all three sizes no longer showed significant effects on any HRV indices after adjusting HR in the models. 

**Figure 2:**
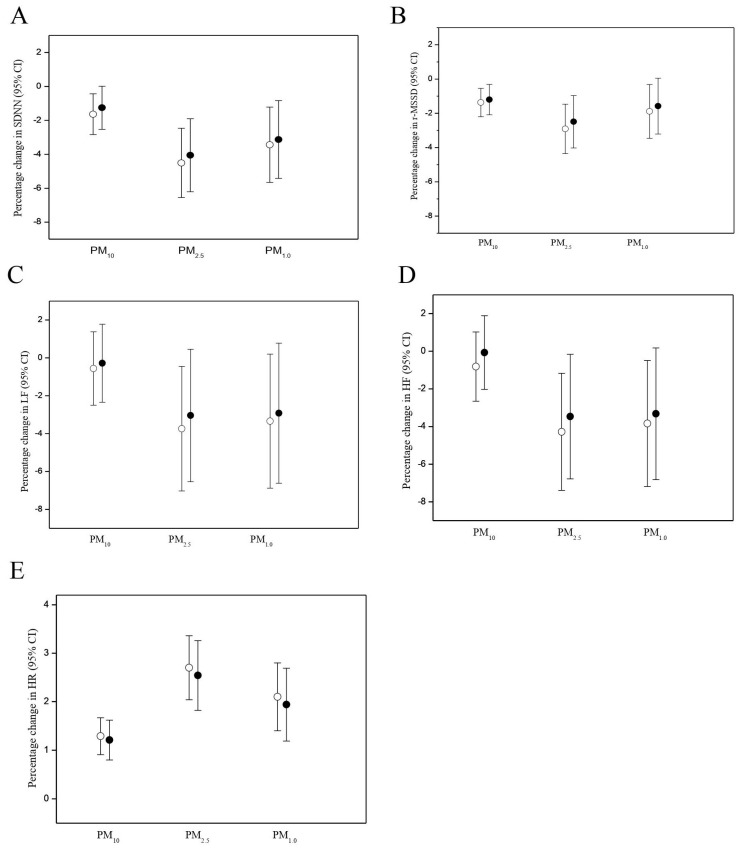
Comparisons of effects of 1-h moving average PM exposure on heart rate variability (HRV) and heart rate (HR) among 54 workers with (solid circles) and without (open circles) adjustment of urinary 8-OHdG levels as estimated using linear mixed effects models. (**A**) Effects of 1-h moving average PM exposure on SDNN with and without adjustment of urinary 8-OHdG levels, (**B**) Effects of 1-h moving average PM exposure on r-MSDD with and without adjustment of urinary 8-OHdG levels, (**C**) Effects of 1-h moving average PM exposure on LF with and without adjustment of urinary 8-OHdG levels, (**D**) Effects of 1-h moving average PM exposure on HF with and without adjustment of urinary 8-OHdG levels, (**E**) Effects of 1-h moving average PM exposure on HR with and without adjustment of urinary 8-OHdG levels. Models were adjusted for age, BMI, smoking, years as a cook, and ambient temperature.

Figure 2 presents the effects of PM exposures on changes in HRV and HR, which are related to pre-workshift urinary 8-OHdG levels. There was no significant difference in the PM effects on HRV and HR in the models with or without adjustment of cooks’ pre-shift urinary 8-OHdG levels. In addition, the difference between the changes in HRV and HR in response to PM exposure in the mixed-effects models with cross-shift changes in urinary 8-OHdG levels did not differ significantly from that in the mixed-effects models without cross-shift changes (data not shown). 

**Figure 3:**
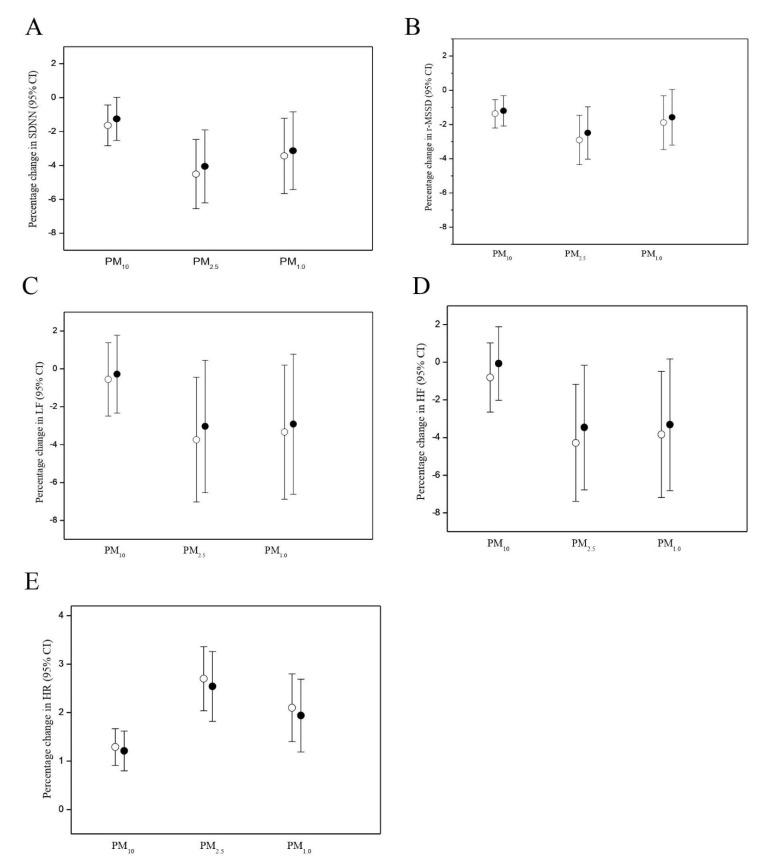
Comparisons of effects of 1-h moving average PM exposure on heart rate variability (HRV) and heart rate (HR) between 26 smokers (solid squares) and 28 non-smokers (open squares) as estimated using the linear mixed effects models. (**A**) Effects of 1-h moving average PM exposure on SDNN with and without cigarette smoking, (**B**) Effects of 1-h moving average PM exposure on r-MSDD with and without cigarette smoking, (**C**) Effects of 1-h moving average PM exposure on LF with and without cigarette smoking, (**D**) Effects of 1-h moving average PM exposure on HF with and without cigarette smoking, (**E**) Effects of 1-h moving average PM exposure on HR with and without cigarette smoking. Models were adjusted for age, BMI, years as a cook, and ambient temperature.

Comparisons of PM exposures on HRV and HR between smokers and non-smokers are shown in Figure 3. Smokers had significantly greater PM_10_-mediated reduction in SDNN, LF, and HF than non-smokers. PM_10_-mediated r-MSSD reduction and HR elevation, however, were not significantly different between smokers and non-smokers. There was no significant difference in PM_2.5_-mediated and PM_1.0_-mediated HRV reduction between smokers and non-smokers either. 

### 3.3. Predictors of Cross-Shift Changes in Urinary 8-OHdG and MDA Levels

**Figure 4:**
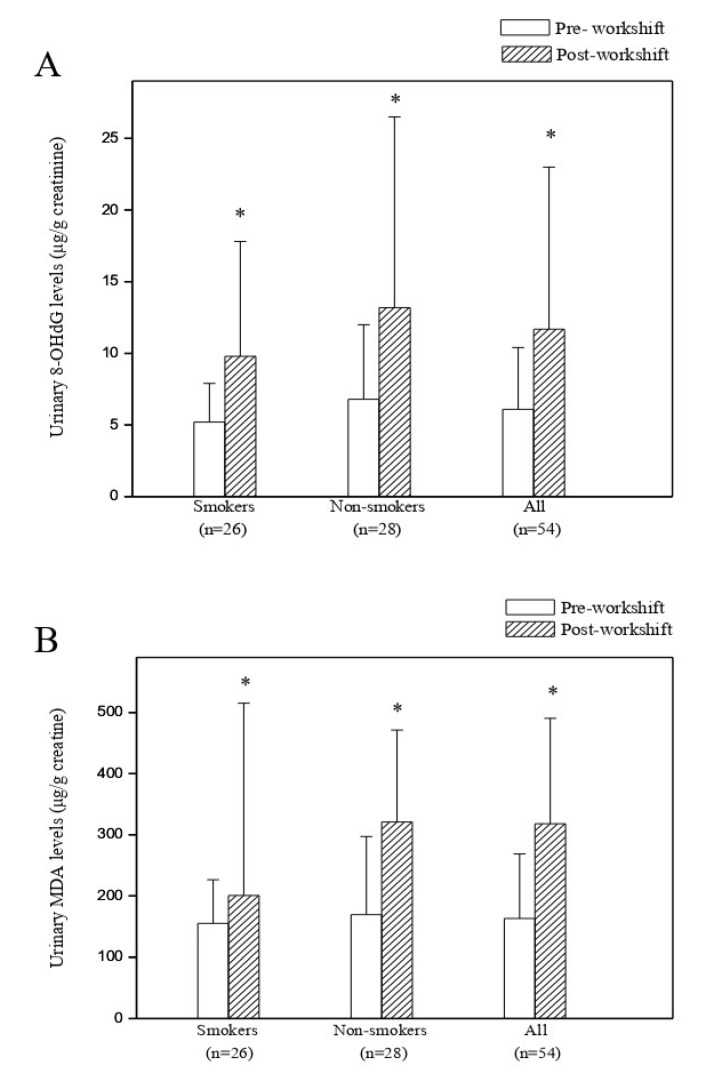
Comparisons of urinary 8-hydroxy-2′-deoxyguanosine (8-OHdG) and malondialdehyde (MDA) levels (mean ± SD (μg/g creatinine)) pre-workshift, and post-workshift among 54 cooks in Chinese restaurants. (**A**) Comparison of urinary 8-OHdG levels between pre-workshift and post-workshift, (**B**) Comparison of urinary MDA levels between pre-workshift and post-workshift. Statistical significance of paired *t-*test for comparing pre- and post-workshift levels after log-transformation at *, *p* < 0.05.

Post-shift urinary levels of 8-OHdG and MDA were consistently higher than the corresponding pre-shift levels, as shown in Figure 4. The student pair *t*-analysis revealed a statistically significant difference between the post-shift urinary 8-OHdG and MDA levels (11.7 ± 11.3 μg/g creatinine, 318.1 ± 172.4 μg/g creatinine, mean ± SD) and pre-shift levels (6.1 ± 4.3 μg/g creatinine, and 163.2 ± 105.6 μg/g creatinine) of the 54 cooks. Differences between pre- and post-shift urinary 8-OHdG and MDA levels remained statistically significant when stratified by smoking status. No statistically significant difference existed between the pre-shift, post-shift or cross-shift changes in 8-OHdG or MDA concentrations of smokers and those of non-smokers. 

As shown in Table 4, cross-shift changes in cooks’ urinary 8-OHdG levels were significantly correlated with PM_2.5_, PM_1.0_, and BaP concentrations but not with PM_10_, pyrene, BkF, Bghip, and DBaeP concentrations in the kitchen, after adjustments had been made for key personal covariates in the models. Only BaP levels were positively correlated with cross-shift changes in cooks’ urinary MDA levels, after adjusting for key personal covariates in the models. PM_10_, PM_2.5_, PM_1.0,_ pyrene, BkF, Bghip, and DBaeP exposures were not associated with cross-shift urinary MAD levels, although their regression coefficients were consistently positive in the analysis. However, age, BMI, cigarette smoking, and years as a cook were not significant predictors for both urinary 8-OHdG and MDA.

## 4. Discussion

This study is, to our knowledge, the first to demonstrate that occupational exposure to PM mass and PAHs in COFs can influence HRV and induce oxidative stress responses among cooks. The main HRV effects are to reduce both time domain indices (SDNN, r-MSDD) and frequency domain indices (LF, HF), in a manner consistent with the effects of ambient PM_2.5_ [28] and 0.02–1 μm particles that have been observed in previous studies [29]. The decrease in SDNN is an indicator of decreased vagal activity or increased sympathetic tone [30], while the decline in r-MSDD and HF components indicates the withdrawal of vagal activity, which is an index of increased cardiovascular events [31,32]. The LF has been previously considered to represent reduced sympathetic activity, but its real physiological interpretation remains debatable [24]. The decrease in total HRV (SDNN) by COFs is one possible reason for the decrease in LF, herein.

The time course of COFs effects on HRV indicates that COFs have both immediate and cumulative effects on cardiac autonomic function since the magnitudes of SDNN, r-MSDD, LF, and HF reduction increase as the averaging interval of PM_2.5_ or PM_1.0_ exposures increases from 15 min to 2 h. The increase in HR may also explain the association of PM with reduced HRV as shown by the fact that elevated PM levels were no longer associated with HRV reduction after adjusting HR in our models. The findings suggest that COFs can influence both the sympathetic and the parasympathetic nervous systems directly, immediately following the exposure [33].

A comparison with a previous study of boilermakers [8] indicates that a given increase in mass concentration of PM_2.5_ induces a larger HRV reduction in cooks than in boilermakers. For example, the SDNN reduction for every 1 mg/m^3^ increase in PM_2.5_ is 62.85% in cooks and 9.39% in boilermakers. The chemical composition of the particles to which workers are exposed may affect the difference between HRV responses. Cooks are occupationally exposed to COFs, which include PAHs, aromatic amines, NPAHs, and aldehydes, while boilermakers are occupationally exposed to metal-rich particles from residual oil fly ash (ROFA) that is generated by the combustion of fuel oil and metal fumes from welding. The difference between the COFs and ROFA contents in the PM may be responsible for the different strengths of the effects of PM on SDNN of cooks and boilermakers. In this study, BMI was significantly and negatively associated with time-domain and frequency-domain HRV indices. This finding was consistent with a study of boilermakers, which also found that obesity may be associated with great susceptibility to the acute cardiovascular effects of fine particles [34]. 

Both of the PM_2.5_ and PM_1.0_ levels were significantly correlated with urinary 8-OHdG levels using linear mixed-effects models in this study. This finding is consistent with three previous studies indicating a significant exposure-response relationship between PM_2.5_ exposure and urinary 8-OHdG levels [35,36,37]. Furthermore, previous studies indicated that submicrometer particles (PM_1.0_) are an environmental stressor, with the potential to induce a series of events by increasing sympathetic activation leading to ischaemia or fatal arrhythmia in high-risk patients with underlying cardiac abnormalities [30]. However, urinary MDA is not associated with PM mass in COFs in the present study since it reacts more readily with other substances in the body than 8-OHdG [38]. The findings, herein, indicate that MDA does not sensitively reflect the oxidative stress in cooks due to exposure to COFs.

This study also reveals a significant exposure-response relationship between particulate BaP in COFs and both of urinary 8-OHdG and MDA levels in Chinese cooks. The metabolism of BaP may damage DNA by the covalent bonding of the metabolite to DNA or by the generation of reactive oxygen species in single-electron redox cycling [39]. Accordingly, Chinese cooks may have an increased risk of developing oxidative DNA injury following exposure to high levels of COFs. Urinary MDA is not associated with PM mass or any PAH component of COFs in the present study since it reacts more readily with other substances in the body than 8-OHdG [40]. The findings, herein, indicate that MDA does not sensitively reflect the oxidative stress in cooks due to exposure to COFs. On the other hand, our multiple linear regression results indicated that obesity did not modify the effects of PM and PAHs on urinary 8-OHdG or MDA levels. In addition, the fact that cook’s pre-exposure 8-OHdG levels did not modify the effect of COFs on HRV in our study indicates that COFs-attenuated HRV reduction may not be affected by individual worker’s oxidative stress. By contrast, the observed differential response of HRV to PM_10_ between smokers and non-smokers implies that cigarette smoking may be an effect modifier of cooks’ autonomic cardiac responses to COFs. 

This study has certain limitations. First, data on other co-pollutants in COFs, such as aldehyde, aromatic amines, and benzene were lacking, possibly confounding the results concerning PM effects. Second, other occupational exposures may affect the post-shift oxidative stress of cooks, such as combustion by products from gas stoves that were not measured in our study. Third, the confounding effects of cigarette smoking could not be totally eliminated. Although smoking was prohibited in the kitchens and dining areas in all restaurants, contamination from cigarette smoke may have occurred if kitchen employees smoked either in the bathrooms or immediately outside kitchen doors. Fourth, personal electrocardiogram monitoring was performed while the subjects worked between 09:00 and 21:00 to obtain data. The available data are still insufficient for adjusting the circadian rhythm (morning, afternoon, evening, and night), since the circadian rhythm may affect the association for PM and HF power of HRV [34]. Additionally, the effects of breathing patterns on HRV and respiration-modulated autonomic activity were not accounted for since they were not measured polysomographically during the monitoring period, these include nasal and mouth airflow, chest wall movement, and abdominal movement. The quantity, periodicity, and timing of vagal cardiac outflow are reportedly related to variations in respiratory depth and respiratory interval in conscious young adults [39]. Regardless of these limitations, our data tend to show that PM in COFs is associated with altered cardiovascular autonomic function and may increase the risk of developing oxidative DNA injury following the exposure of Chinese cooks to high levels of COFs.

## 5. Conclusions

Reduced heart rate variability (HRV) is an indicator of cardiac autonomic dysfunction, and an independent predictor of cardiovascular mortality—particularly sudden cardiac death and arrhythmias. It has been associated with a short-term exposure to ambient and occupational particulate matter (PM). Cooks are occupationally exposed to cooking oil fumes (COFs) of which PM and polycyclic aromatic hydrocarbons (PAHs) are important components. Carcinogenic PAHs can generate reactive oxygen species (ROS) via cytochrome P-450 A1 during the metabolic process. Urinary 8-hydroxy-2′-deoxyguanosine (8-OHdG), a biomarker of oxidative stress on DNA, can be used to evaluate the extent of repair of ROS-induced DNA damage in both clinical and occupational fields. This study investigates the effects of PM mass and PAHs in COFs on 8-OHdG and of PM mass in COFs on HRV. It also explores whether pre-exposure baseline oxidative stress levels modified the effects of exposure to PM on HRV. Exposure to PM in COFs from 15 min to 2 h were associated with HRV reduction and heart rate increase among cooks. Cooks’ urinary 8-OHdG levels were significantly elevated after a work-shift exposure of COFs. The levels of particulate benzo(a)pyrene in COFs were positively correlated with cross-workshift urinary 8-OHdG levels. Cross-workshift urinary 8-OHdG levels did not modify the effects of COFs on HRV. Exposure to COFs can cause disturbed autonomic function and an increase the risk of developing oxidative DNA injury among cooks in Chinese restaurants.

## Figures and Tables

**Table 1:** Personal characteristics and environmental exposures of cooks (*n* = 54).

Characteristics	Mean ± SD	Median	Range
Age (years)	33.6 ± 10.5	34.5	15–56
Body mass index (kg/m^2^)	23.2 ± 3.7	22.1	17.6–32.9
Years as a cook (years)	13.4 ± 10.5	14.0	1–40
Obese subjects ^†^	10 (20.8) *	─	─
Cigarette smokers	26 (48.1%) *	─	─
Heart rate (5-min mean, beats/minute)	91.3 ± 10.2	90.4	64–115
PM_10_ (5-min mean, μg/m^3^)	72.8 ± 134.7	45.6	1.9–2481.9
PM_2.5_ (5-min mean, μg/m^3^)	49.7 ± 56.2	35.9	1.5–1168.3
PM_1.0_ (5-min mean, μg/m^3^)	37.3 ± 36.2	28.4	1.2–532.3
Pyrene (ng/m^3^)	4.5 ± 8.9	3.8	ND–58.7
Benzo(k)fluroranhene (ng/m^3^)	1.7 ± 3.0	1.4	ND–25.3
Benzo(a)pyrene (ng/m^3^)	11.4 ± 20.1	6.9	0.1–154.4
Benzo(ghi)perylene (ng/m^3^)	8.8 ± 20.8	5.5	ND–177.7
Dibenzo(a,e)pyrene (ng/m^3^)	9.3 ± 24.6	6.1	ND–179.2
Ambient temperature (5-min mean, °C)	25.8 ± 3.9	26.4	16.0–36.9

SD: Standard deviation; ND: Measurements of samples are below the detection limit of the analytical method for PAHs; ND was calculated as half of the detection limit of the analytical method. The numbers (proportion) of ND samples for Pyrene, Benzo(k)fluoranthene, Benzo(ghi)perylene, and Dibenzo(a,e)pyrene were 2 (3.7%), 1 (1.9%), 2 (3.7%), and 5 (9.3%), respectively. * N (%). ^†^ Obese subjects: BMI ≥ 27 (a criteria adopted by the Department of Health, Taiwan).

**Table 2:** Estimated percentage change (95% CIs) in HR and time-domain HRV indices by an interquartile range (IQR) ^†^ increase in PM exposure (*n* = 54) ^‡^.

Outcome	Moving Average Period	PM_10_	PM_2.5_	PM_1.0_
HR				
	15-min	0.71 (0.42 to 1.01) *	1.37 (0.91 to 1.82) *	1.26 (0.72 to 1.80) *
	30-min	1.55 (0.80 to 1.51) *	2.39 (1.79 to 3.01) *	1.92 (1.26 to 2.59) *
	1-h	1.29 (0.91 to 1.67) *	2.70 (0.53 to 4.91) *	2.10 (1.41 to 2.81) *
	2-h	1.16 (0.41 to 1.92) *	2.17 (1.12 to 3.23) *	1.26 (0.17 to 2.36) *
SDNN				
	15-min	−0.90 (−1.78 to −0.02) *	−2.19 (−3.52 to −0.85) *	−1.78 (−3.36 to −0.16) *
	30-min	−1.41 (−2.46 to −0.35) *	−4.27 (−5.99 to −2.51) *	−3.20 (−5.10 to −1.27) *
	1-h	−1.63 (−2.83 to −0.49) *	−4.51 (−6.53 to −2.45) *	−3.44 (−5.63 to −1.20) *
	2-h	−0.14 (−2.85 to 2.63)	−3.85 (−7.41 to −0.15) *	−3.92 (−7.52 to −0.18) *
r-MSSD				
	15-min	−0.80 (−1.42 to −0.18) *	−0.76 (−1.29 to −0.23) *	−0.72 (−1.18 to −0.26) *
	30-min	−1.24 (−1.85 to −0.63) *	−2.33 (−3.60 to −1.05) *	−1.72 (−3.01 to −0.43) *
	1-h	−1.37 (−2.21 to −0.53) *	−2.91 (−4.34 to −1.46) *	−1.89 (−3.45 to −0.31) *
	2-h	−0.61 (−2.53 to 1.34)	−3.26 (−5.34 to −1.13) *	−3.45 (−6.04 to −0.79) *

^†^ The IQRs of moving average for 15-min, 30-min, 1-h, and 2-h in PM_10_ were 64.39, 60.83, 60.70, and 62.60 μg/m^3^, respectively. The IQRs of moving average for 15-min, 30-min, 1-h, and 2-h in PM_2.5_ were 43.03, 46.71, 46.64, and 41.07 μg/m^3^, respectively. The IQRs of moving average for 15-min, 30-min, 1-h, and 2-h in PM_1.0_ were 33.29, 33.58, 33.75, and 29.54 μg/m^3^, respectively. ^‡^ Models were adjusted for age, BMI, smoking, years as a cook, and ambient temperature.* *p* < 0.05.

**Table 3:** Estimated percentage change (95% CIs) in LF and HF of frequency domain HRV indices by an interquartile range (IQR) ^†^ increase in PM exposure (*n* = 54) ^‡^.

Outcome	Moving Average Period	PM_10_	PM_2.5_	PM_1.0_
LF				
	15-min	−0.27 (−1.63 to 1.12)	−0.71 (−0.98 to −0.44) *	−1.58 (−2.84 to −0.30) *
	30-min	−0.45 (−2.27 to 1.41)	−2.66 (−4.86 to −0.41) *	−2.73 (−4.87 to −0.54) *
	1-h	−0.56 (−2.48 to 1.40)	−3.69 (−6.92 to −0.34) *	−3.34 (−6.25 to −0.34) *
	2-h	−1.45 (−5.64 to 2.94)	−6.51 (−11.94 to −0.74) *	−6.05 (−11.58 to −0.18) *
HF				
	15-min	−0.49 (−1.56 to 0.60)	−1.31 (−2.30 to −0.31) *	−1.47 (−2.69 to −0.23) *
	30-min	−0.62 (−2.21 to 1.00)	−3.00 (−5.35 to −0.58) *	−2.77 (−5.04 to −0.44) *
	1-h	−0.81 (−2.63 to 1.05)	−4.28 (−7.34 to −1.17) *	−3.84 (−7.13 to −0.43) *
	2-h	−1.20 (−5.27 to 3.03)	−6.86 (−12.09 to −1.33) *	−6.64 (−11.22 to −1.82) *

^†^ The IQRs of moving average for 15-min, 30-min, 1-h, and 2-h in PM_10_ were 64.39, 60.83, 60.70, and 62.60 μg/m^3^, respectively. The IQRs of moving average for 15-min, 30-min, 1-h, and 2-h in PM_2.5_ were 43.03, 46.71, 46.64, and 41.07 μg/m^3^, respectively. The IQRs of moving average for 15-min, 30-min, 1-h, and 2-h in PM_1.0_ were 33.29, 33.58, 33.75, and 29.54 μg/m^3^, respectively. ^‡^ Models were adjusted for age, BMI, smoking, years as a cook, and ambient temperature. * *p* < 0.05.

**Table 4:** Predictors of cross-shift changes in urinary 8-OHdG and MDA levels in 54 cooks using linear mixed-effect regression models ^a^.

	Log_10_ 8-OHdG (μg/g Creatinine)	Log_10_ MDA (μg/g Creatinine)
Predictors	Regression coefficient (95% Confidence interval)	Regression coefficient (95% Confidence interval)
Log PM_10_	2.842 (−2.672 to 8.356)	0.539 (−5.581 to 6.658)
Log PM_2.5_	2.080 (0.869 to 3.291) ^b^	2.305 (−10.565 to 15.175)
Log PM_1.0_	2.574 (1.162 to 3.986) ^b^	1.114 (−6.249 to 8.477)
Log_10_ Pyrene	0.168 (−0.107 to 0.442)	0.138 (−0.167 to 0.443)
Log_10_ Benzo(k)fluoranthene	1.480 (0.245 to 2.715)	0.403 (−0.968 to 1.774)
Log_10_ Benzo(a)pyrene	0.088 (0.055 to 0.122) ^b^	0.078 (0.044 to 0.113) ^b^
Log_10_ Benzo(ghi)perylene	0.077 (−0.816 to 0.970)	0.057 (−0.934 to 1.048)
Log_10_ Dibenzo(a,e)pyrene	0.089 (−0.025 to 0.203)	0.111 (−0.016 to 0.237)

^a^ Models adjusted for age, body mass index, and cigarette smoking. MDA: Malondialdehyde; 8-OHdG: 8-Hydroxy-29-deoxyguanosine.^b^ *p* < 0.05.

## Data Availability

Data is contained within the article.
